# Spontaneous rectus sheath and retroperitoneal haemorrhage secondary to enoxaparin injections

**DOI:** 10.1093/jscr/rjad482

**Published:** 2023-08-23

**Authors:** Aroon Sohail, Jawad Naqvi, Paul Wilson

**Affiliations:** Lancaster Medical School, Lancaster University, Lancaster LA1 4YW, United Kingdom; Department of General Surgery, Royal Lancaster Infirmary, Lancaster LA1 4RP, United Kingdom; Department of Radiology, Royal Lancaster Infirmary, Lancaster LA1 4RP, United Kingdom; Department of General Surgery, Royal Lancaster Infirmary, Lancaster LA1 4RP, United Kingdom

**Keywords:** general surgery, vascular, interventional radiology, general radiology, gastrointestinal, haemorrhage

## Abstract

Bleeding from enoxaparin is a recognized side effect; however, the formation of rectus sheath and retroperitoneal haematomas is a rare and potentially life-threatening complication. Patients may present insidiously and without symptoms; therefore, high clinical suspicion of a bleeding intra-abdominal haematoma should be suspected in patients with a combination of clinical and biochemical evidence of bleeding. Treatment is patient dependent and is either interventional or conservative in approach. Clinicians should be mindful when prescribing high-dose enoxaparin with further caution advised for at-risk patients.

## INTRODUCTION

Enoxaparin is a low molecular weight heparin, routinely used for the prophylaxis or treatment of venous thromboembolism (VTE). Retroperitoneal and rectus sheath haematomas are rare complications of enoxaparin injections, leading to intra-abdominal haemorrhage, which could be life threatening. Herein, we present a case of rectus sheath and retroperitoneal haematoma formation with active haemorrhage secondary to enoxaparin injections.

## CASE REPORT

A 79-year-old woman on the medical ward was complaining of severe abdominal pain overnight. She was admitted 1 week prior with coronavirus disease 2019 (COVID-19) pneumonitis and was being treated with remdesivir and dexamethasone. She was on a high-dose subcutaneous enoxaparin regime (1 mg/kg BD) as per local guidelines for VTE prophylaxis. She had a background of neurofibromatosis type-1 (NF1) and osteoarthritis.

The patient described the abdominal pain starting several days prior but denied any acute injury or event as a potential trigger. The patient was pale with cold peripheries and a prolonged central capillary refill time. Her vital signs showed a hypotension of 90 systolic and a heart rate of 85 beats per minute. There were multiple visible suprapubic bruising sites indicative of enoxaparin injections. On palpation, there was a small mass posterior to the area of bruising with a separate and much larger mass in the left iliac fossa. There was no evidence of external bleeding.

Blood results are illustrated in Table 1, which compare results on assessment to 3 days prior. Significant abnormalities included an acute fall in haemoglobin to 67 g/l, an increase in the white blood cell count to 24.5 × 10^9^/l and a two-fold increase in both urea and creatinine with a reduction in the estimated glomerular filtration rate indicating an acute kidney injury.

**Table 1:** Laboratory test results on assessment compared to 3 days prior

Test	Results	Results 3 days prior	Reference range
Haemoglobin	67	120	115–165 g/l
Platelets	372	361	150–400 × 10^9^/l
White blood cells	24.5	8.9	4.0–10.0 × 10^9^/l
Neutrophils	18	4.5	2.0–7.5 × 10^9^/l
C-Reactive protein	24	<5	0.0–5.0 mg/l
Sodium	135	140	133–146 mmol/l
Potassium	4.3	3.7	3.5–5.3 mmol/l
Urea	17.1	7.8	2.5–7.8 mmol/l
Creatinine	96	43	45–84 μmol/l
Estimated glomerular filtration rate	49	>90	>90 ml/min/1.73 m^2^
International normalized ratio	1	n/a	1–1.3
Activated partial thromboplastin time ratio	1.02	n/a	0.84–1.16
Fibrinogen	4.9	n/a	1.5–4.5 g/l

**Figure 1:**
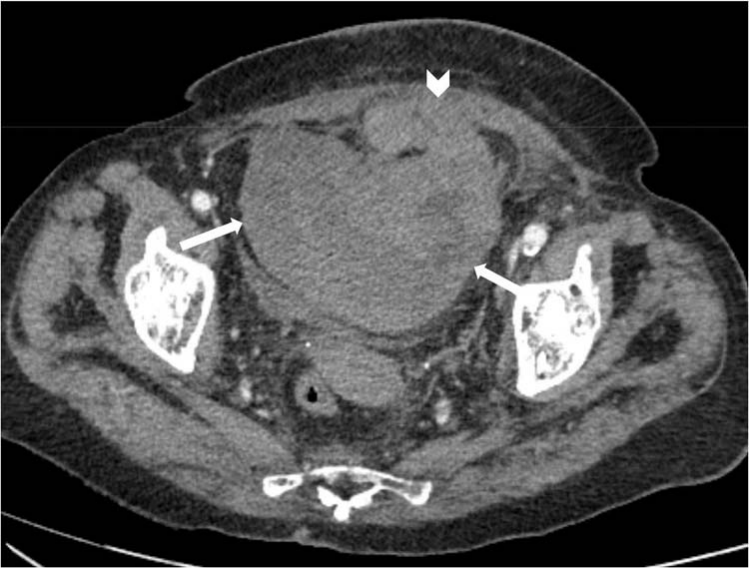
Axial non-contrast CT abdomen and pelvis at the level of acetabulum. Rectus sheath haematoma (chevron) that appears to have dissected through the rectus sheath into the extraperitoneal pelvic space (arrows).

**Figure 2:**
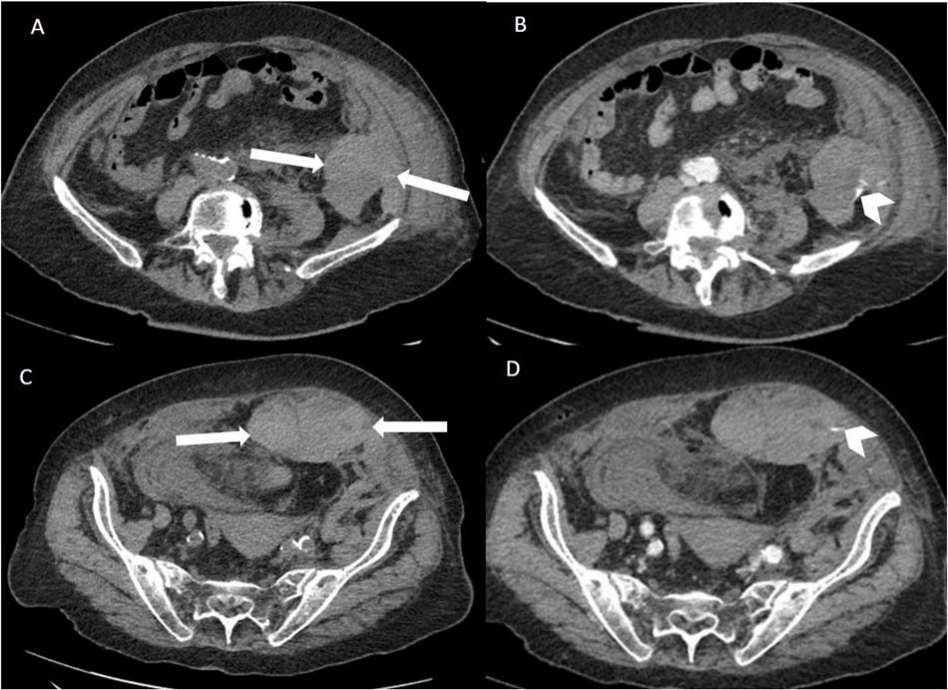
Axial non-contrast and post-contrast (100-s delay) CT abdomen and pelvis slices demonstrating two further left abdominal wall and pre-peritoneal collections (white arrows) with high attenuation on non-contrast slices (**A** and **C**) in keeping with haematoma. There is contrast blush in two locations (white chevrons in **B** and **D**) in keeping with active bleeding. The vascular territories that have likely contributed to bleeding include branches of left inferior epigastric and left lumbar arteries.

Suspecting internal haemorrhage, contrast-enhanced computed tomography (CECT) of the abdomen and pelvis was arranged. The CT images showed the presence of multiple haematomas with evidence of active bleeding (Figs 1 and 2).

After discussions between the interventional radiologist, general surgeon and haematologist, a conservative approach to management was taken. The patient’s old age, moderate frailty and comorbidities favoured conservative treatment, with an interventional approach to be considered if conservative measures were unsuccessful. A total of 6 units of packed red blood cells were transfused over the course of 5 days. Tranexamic acid was also administered during the same time period at a regime of 1 g three times a day. Empirical antibiotic cover was also given. As there was no coagulopathy and a significant interval since the last enoxaparin injection, no reversal agent nor coagulation factors were given. After monitoring for 5 days, the patient’s haemoglobin normalized to 121 g/l. She was subsequently discharged following 5 further days. No routine follow-up was required.

## DISCUSSION

Haematomas can be classified according to their location, for example, abdominal, pelvic and intracranial. Abdominal haematomas are further sub-grouped into abdominal wall or intra-abdominal. Abdominal wall haematomas develop secondary to bleeding within the abdominal muscle wall, specifically the rectus sheath due to its highly vascularized anatomy. The most common vascular territory for rectus sheath haematoma (RSH) are the inferior and superior epigastric arteries and veins as they supply the superficial tissue and muscles of the abdominal wall [1, 2].

As with any other form of haemorrhage, the likelihood of developing a RSH is multifactorial with risk factors including trauma, frailty, age, gender, coagulopathy and anticoagulation medications. In the majority of cases, the RSH is localized to the abdominal wall; however, there is the potential for extension of the haematoma either supraumbilical or contralaterally with increased risk of transformation into an intra-abdominal haematoma particularly in patients with connective tissue disorders [3].

Retroperitoneal haematomas (RPH) form due to injury of the vascular or parenchymal tissue within the retroperitoneal space [4]. Retroperitoneal haematomas are sub-grouped depending on their aetiology, divided into traumatic and non-traumatic origins. Traumatic origins would include blunt or penetrating trauma whereas non-traumatic would include iatrogenic or spontaneous causes of haemorrhage.

The most common symptom that presents with a RSH is abdominal pain, typically exacerbated by movement or palpation. There may or may not be a palpable mass or visible bruising on the skin surface. RPH may present insidiously, often being missed with signs and symptoms apparent only after a significant quantity of blood loss. If a haematoma is suspected, a comprehensive physical examination should be conducted to identify signs and symptoms suggestive of occult blood loss including hypotension, tachycardia, cold peripheries and pallor.

Blood tests should be conducted to monitor several parameters including haemoglobin level to determine the degree of haemorrhage, coagulation profile to identify any underlying coagulopathy, liver function tests to determine any underlying hepatic dysfunction, infective markers to monitor evidence of infection and a renal profile to determine any secondary kidney injury [5].

The optimal modality of imaging for a suspected actively bleeding haematoma is a CECT. This will provide crucial details relating to the haematoma including size, location, consistency and involvement of surrounding structures. A blush of contrast within the haematoma would indicate active bleeding, necessitating urgent intervention [6, 7].

The management of an actively bleeding haematoma is multi-faceted and should incorporate a multi-disciplinary team (MDT) approach including input from interventional radiology, general surgery and haematology. A minimally invasive approach may involve artery embolization by interventional radiology, whereas an invasive surgical approach could involve a laparotomy and haematoma evacuation with ligation of the bleeding vessel. A conservative approach would include blood transfusion and anti-fibrinolytic medication [3, 4]. Regardless, a primary survey should always be the first-line approach with wide bore intravenous access secured with fluid and blood resuscitation, cessation of any anticoagulation medications and coagulopathy reversal if indicated [8, 9].

Our patient likely developed her abdominal wall/extraperitoneal haematomas secondary to the subcutaneous enoxaparin injections. She was on 40 mg twice-a-day regime as per local guidelines for VTE prophylaxis for COVID-19 positive patients. She was visibly frail with a low body fat percentage, increasing the risk for injury to the vascular structures associated with the abdominal wall.

Furthermore, our patient had a background of NF1, which multiple studies and previous case reports have demonstrated increases the risk for vascular complications [10]. Increased vessel and surrounding tissue fragility and impaired haemostasis secondary to vein wall infiltration by the neurofibroma are proposed mechanisms underlying the vascular compromise [11–13].

Hazenberg *et al*. noted in a case series 12 patients who had developed either RSH or RPH secondary to enoxaparin injection in COVID-19 positive patients [14]. Their study identified patients who were predominantly on therapeutic dose enoxaparin and likely developed RPH/RSH due to an extended period of time receiving anticoagulation. Additional risk factors suggested included abdominal wall injection, steroid therapy, coughing and old age. Their study suggested a mortality rate of 70% in COVID-19 related RPH, significantly higher than the non-COVID-19 rate of around 10% [15, 16]. The association of enoxaparin injections for patients who are COVID-19 positive and developing haematoma complications is a newly reported phenomenon and is associated with a significantly raised mortality rate.

## CONCLUSION

Abdominal haematomas secondary to subcutaneous enoxaparin injections are a rare but recognized complication. These normally take the form of rectus sheath or retroperitoneal haematomas due to the subsequent injury of the underlying vascular structures. Patients may present with abdominal pain but can be asymptomatic. Suspicion of an acutely bleeding haematoma requires CT imaging and an MDT discussion with relevant specialties to determine if an interventional or conservative approach is appropriate. Clinicians should be mindful when prescribing high-dose subcutaneous enoxaparin with caution advised for at-risk patients including old age, frailty, relevant comorbidities, steroid therapy, COVID-19 and underlying coagulopathies.

## CONFLICT OF INTEREST STATEMENT

Authors declare that they have no competing interests.

## FUNDING

The authors received no funding for this work.

## AUTHORS' CONTRIBUTIONS

A.S.: conceptualization, investigation, writing—original draft, corresponding author. J.N.: supervision, writing—review and editing, data curation. P.W.: supervision, writing—review and editing.

## DATA AVAILABILITY

Data sharing is not applicable to this article as no new data were created or analysed in this study.

## CONSENT FOR PUBLICATION

Written informed consent was obtained from the patient for publication of this case report and accompanying images.

## ETHICAL APPROVAL

Ethical approval was provided/waived by the authors’ institution.

## PROVENANCE AND PEER REVIEW

Not commissioned, externally peer reviewed.
